# Supplementary material for: Higher loss of livelihood and impoverishment in households affected by tuberculosis compared to non-tuberculosis affected households in Zimbabwe: a cross-sectional study
Source: medRxiv. 2023 Dec 5:2023.12.05.23299470. Preprint. [Version 1] doi: 10.1101/2023.12.05.23299470 (PMC10723493; doi:10.1101/2023.12.05.23299470)
Supplement: Supplement 1 [file NIHPP2023.12.05.23299470v1-supplement-1.pdf]

medRxiv preprint doi: https://doi.org/10.1101/2023.12.05.23299470; this version posted December 5, 2023. The copyright holder for this preprint (which was not certified by peer review) is the author/funder, who has granted medRxiv a license to display the preprint in perpetuity.
It is made available under a CC-BY 4.0 International license .

Supplement table 1: Indicators contributing to the livelihood variable.

Livelihood capital	Indicators
Financial capital	• Sale of assets.‡<br>• Spending savings.‡<br>• Failure to repay loans.<br>• Pledging future crops/cattle/livestock‡<br>• Borrowing at exorbitant interest rates‡<br>• Reduction in household income.
Physical capital	• Sale of productive assets.‡<br>• Failure to replace productive assets.
Social capital	• Changes in relationship with neighbours for the worse.<br>• Changes in relationship with family members for the worse.<br>• Received support from neighbours/family.
Natural capital	• Reduction in land that is farmed (idle land/leasing).<br>• Sale of land/cattle to finance TB.
Human capital	• Change in head of household (death/abandonment by spouse/family).<br>• Physical debility resulting in loss of labour for household.<br>• Insufficient food.<br>• Education of children affected†

†=A derived variable obtained from either moving children to cheaper schools and/or withdrawing children from school; ‡=Coping strategies.

medRxiv preprint doi: https://doi.org/10.1101/2023.12.05.23299470; this version posted December 5, 2023. The copyright holder for this preprint (which was not certified by peer review) is the author/funder, who has granted medRxiv a license to display the preprint in perpetuity.
It is made available under a CC-BY 4.0 International license .

Supplementary Figure 1: Study sites

medRxiv preprint doi: https://doi.org/10.1101/2023.12.05.23299470; this version posted December 5, 2023. The copyright holder for this preprint (which was not certified by peer review) is the author/funder, who has granted medRxiv a license to display the preprint in perpetuity.
It is made available under a CC-BY 4.0 International license .

Supplementary file: Creating a binary variable for loss of livelihood

Use of a sustainable livelihood framework-based measure to create a dichotomous outcome variable for loss of livelihood (Yes/No).

Authors and institutions

Collins Timire, [1-3] Rein Houben, [4] Debora Pedrazzoli, [4] Rashida Abbas Ferrand, [1,3] Claire Calderwood, [1,3] Virginia Bond, [5,6] Fredrick Mbiba, [3] Katharina Kranzer [1,3,7,8]

[1] Department of Clinical Research, London School of Hygiene & Tropical Medicine, London, UK

[2] AIDS & TB Department, Ministry of Health and Child Care, Harare, Zimbabwe

[3] The Health Research Unit, Biomedical Research & Training Institute, Harare, Zimbabwe

[4] Department of Infectious Disease Epidemiology, London School of Hygiene and Tropical Medicine, UK

[5] Department of Global Health and Development, Faculty of Public Health and Policy LSHTM, London, UK

[6] Social Sciences Unit, Zambart, Lusaka, Zambia

[7] Division of Infectious Diseases and Tropical Medicine, University Hospital, LMU Munich

[8] German Center for Infection Research (DZIF), partner site Munich

Corresponding author details:
Collins Timire
London School of Hygiene & Tropical Medicine, UK
Clinical Research Department, Keppel Street, Bloomsbury, London WC1E 7HT, UK
Collins.Timire@LSHTM.ac.uk +263777 687328 /+447719721145

Alternative corresponding author details:
Katharina Kranzer
London School of Hygiene & Tropical Medicine, UK
Clinical Research Department, Keppel Street, Bloomsbury, London WC1E 7HT, UK
Katharina.Kranzer@LSHTM.ac.uk +263774795737

medRxiv preprint doi: https://doi.org/10.1101/2023.12.05.23299470; this version posted December 5, 2023. The copyright holder for this preprint (which was not certified by peer review) is the author/funder, who has granted medRxiv a license to display the preprint in perpetuity.
It is made available under a CC-BY 4.0 International license .

Household questionnaire

The household questionnaire was interviewer administered to heads of households in 180 TB affected and in 90 non-affected households. The questionnaire was administered in local language by CT (the PI) and FM (Research Assistant). The development of the questionnaire was informed by the variables in the patient cost surveys, the five capital assets on the sustainable livelihood framework, the pilot study in HIV programming in Zimbabwe, and researchers' field experiences in Zimbabwe. Some of which were derived during analysis. Data from the paper-based questionnaires were entered in EpiData entry software for cleaning and were exported to Stata version 13.0 (StataCorp, College Station, TX, USA) for analysis.

Variables in the household questionnaire

The outcome variable, loss of livelihood was derived from five capital assets. Variables were derived from each of the five capital assets. The variables that were included in the model are listed below.

Table S1: Variables included in the model to come up with an indicator for loss of livelihood

Capital asset	Definition	Questions	Variable name	Coding
Human	Ability to work now or in the future and acquire skills	i. Was child transferred to a cheaper school?	N/A	[Yes=1/No=0]
		ii. Was child withdrawn from school?	N/A	
		iii. Was education of child affected? [*derived from i and ii*]	educaffect	
		iv. During the past month, did you worry that your household had insufficient food?	insufood	[Yes=1/No=0]
Financial	Refers to cash, stock and savings. It may take the form of savings, earnings (whether regular wages or one-off payments), access to loans, or money stored in saleable property such as livestock.	i. Did your household spend savings?	spendsaving	[Yes=1/No=0]
		ii. Did your household borrow some money	borrow	[Yes=1/No=0]
		iii. Did your household pledge future crops or livestock?	pledged	[Yes=1/No=0]
		iv. Did your household fail to repay loans?	failrepay	[Yes=1/No=0]
		v. Income of household 12 months prior the interview	N/A	
		vi. Income of household at the time of interview	N/A	
		household income loss [*derived from v and vi*]	hhincomlos	[Yes=1/No=0]
Physical	Refers to assets such as buildings, machinery and equipment.	i. Did your household sell any assets?	soldassets	[Yes=1/No=0]
Social	Are relations which are based on exchange, trust and reciprocity that households depend on.	i. Relationship with family 12 months before interview on a scale of [0-10], where 0 is worst, and 10 is excellent]	N/A	N/A
		ii. Relationship with family at the time of interview on a scale of [0-10], where 0 is worst, and 10 is excellent]	N/A	N/A
		iii. Change in relations with family for the worse [derived variable from i and ii]	frelchange	[Yes=1/No=0]
		iv. Relationship with neighbours 12 months before interview on a scale of [0-10], where 0 is worst, and 10 is excellent]	N/A	N/A
		v. Relationship with neighbours at the time of interview on a scale of [0-10], where 0 is worst, and 10 is excellent]	N/A	N/A
		vi. Change in relations with neighbours for the worse [*derived from i & ii*]	nrelchange	[Yes=1/No=0]
		vii. Did your household receive social support (food/ cash?)	socspt	[Yes=1/No=0]
Natural	Include land and livestock	i. Did your household sell productive assets?	sold assets	[Yes=1/No=0]
		ii. Did your household fail to replace the productive assets that were sold?	failrestock	[Yes=1/No=0]

medRxiv preprint doi: https://doi.org/10.1101/2023.12.05.23299470; this version posted December 5, 2023. The copyright holder for this preprint (which was not certified by peer review) is the author/funder, who has granted medRxiv a license to display the preprint in perpetuity.
It is made available under a CC-BY 4.0 International license .

Creating a binary variable for loss of livelihood (Yes/No) from the 13 variables.

First, a global list of all the 13 variables was created using the syntax below.
global xlist1 soldassets hhincomlos insufood spendsaving borrow failrepay prodasset educaffect pledged socspt nrelchange frelchange
global id uniqueid

Second, the variables were correlated. Since the variables are binary (Yes/No), the tetrachoric approach was applied. The correlations were presented in a correlation matrix (**Table S1**).

Correlations among variables
tetrachoric $xlist1, posdef
adj-corr soldassets hhincomloss spendsavingg prodasset educaffect pledged failrepay insufood borrow failrestock frelchangee nrelchange socspt

Table S1: Correlation matrix of the variables included in the factor analysis.

	soldassets	hhincomloss	insufood	spendsaving	Borrow	failrepay	prodasset	eduaffect	pledge	socspt	nrelchange	frelchange	failrestock
soldassets	1.00												
hhincomloss	0.4458	1.00											
insufood	0.3337	0.4188	1.00										
spendsaving	0.1097	0.3449	0.0420	1.00									
borrow	0.4254	0.3508	0.2906	0.3424	1.00								
failrepay	0.4403	0.4707	0.4959	0.2173	0.8812	1.00							
prodasset	0.3952	0.1733	0.2170	0.1473	0.0735	0.0951	1.00						
eduaffect	0.4802	0.4274	0.4322	0.1629	0.4443	0.4953	0.1778	1.00					
pledge	0.4017	0.8477	0.1225	0.2995	0.3999	0.4087	0.2829	0.0534	1.00				
socspt	-0.0684	-0.1206	-0.0748	-0.0983	0.0894	0.0052	0.1186	-0.1858	0.0740	1.00			
nrelchange	0.4024	0.4656	0.0925	0.2216	0.2116	0.4529	0.2365	0.3718	0.3130	-0.3065	1.00		
frelchange	0.3294	0.2734	0.0573	0.1675	0.1873	0.4171	-0.0017	0.4196	0.0640	0.0322	0.5987	1.00	
failrestock	0.3748	0.1685	0.1930	0.1449	0.0403	0.0696	0.9930	0.1812	0.2632	0.0986	0.2896	0.0295	1.00

medRxiv preprint doi: https://doi.org/10.1101/2023.12.05.23299470; this version posted December 5, 2023. The copyright holder for this preprint (which was not certified by peer review) is the author/funder, who has granted medRxiv a license to display the preprint in perpetuity.
It is made available under a CC-BY 4.0 International license .

Measure of sampling adequacy

The Kaiser-Meyer-Olkin (KMO) was used as the measure of sampling adequacy. The following syntax was used.

pca $xlist1, components (1)
screeplot, yline(1) ci(het)
estat kmo

pca $xlist1, components (1)
Principal components correlation

Number of obs	267
Number of comp	1
Trace	13
Rho	0.2293

Component	Eigenvalue	Difference	Proportion	Cumulative
Comp1	2.98083	1.11858	0.2293	0.2293
Comp2	1.86225	0.677627	0.1433	0.3725
Comp3	1.18463	0.103454	0.0911	0.4637
Comp4	1.08117	0.0321132	0.0832	0.5468
Comp5	1.04906	0.127538	0.0807	0.6275
Comp6	0.921523	0.0336749	0.0709	0.6984
Comp7	0.887848	0.153076	0.0683	0.7667
Comp8	0.734772	0.0480941	0.0565	0.8232
Comp9	0.686678	0.0714222	0.0528	0.8761
Comp10	0.615256	0.0875471	0.0473	0.9234
Comp11	0.527709	0.0981294	0.0406	0.9640
Comp12	0.429579	0.390895	0.0330	0.9970
Comp13	0.0386842	.	0.0030	1.0000

Principal components (eigenvectors)

Variable	Comp1	Unexplained
soldassets	0.3569	0.6202
hhincomloss	0.3187	0.6972
spendsaving	0.1883	0.8943
prodasset	0.2859	0.7564
educaffect	0.3131	0.7077
pledged	0.1739	0.9098
failrepay	0.3695	0.5929
insufood	0.2272	0.8461
borrow	0.3062	0.7205
failrestock	0.2843	0.7591
frelchange	0.2450	0.8211
nrelchange	0.3169	0.7006
socspt	-0.0474	0.9933

medRxiv preprint doi: https://doi.org/10.1101/2023.12.05.23299470; this version posted December 5, 2023. The copyright holder for this preprint (which was not certified by peer review) is the author/funder, who has granted medRxiv a license to display the preprint in perpetuity.
It is made available under a CC-BY 4.0 International license .

Screeplot

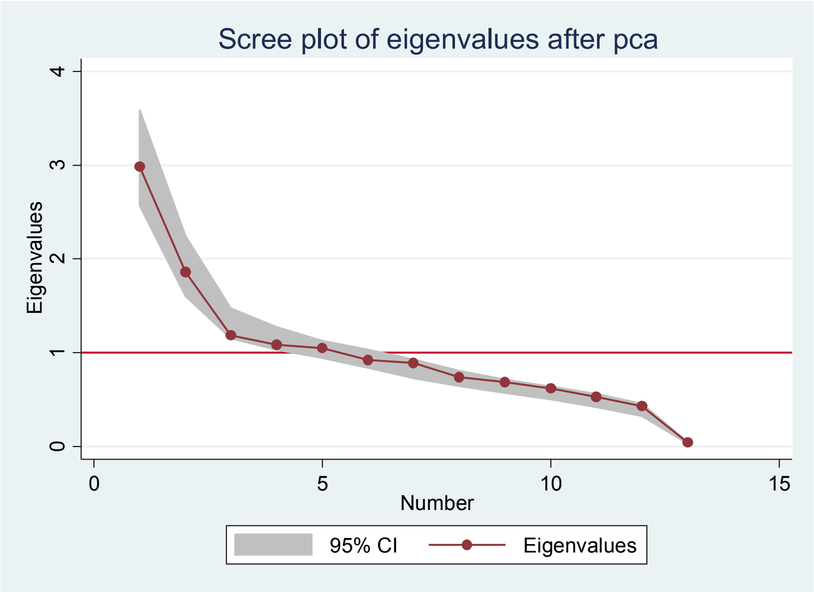

The KMO measure shows that xxxxxxxxxxxxxx

**Variable**	**KMO**
soldassets	0.8621
hhincomlos	0.7968
spendsaving	0.7098
prodasset	0.5313
educaffect	0.8401
pledged	0.7655
failrepay	0.7223
insufood	0.7395
borrow	0.6967
failrestock	0.5296
frelchange	0.6947
nrelchange	0.7017
socspt	0.4720
**Overall**	**0.6588**

**KMO measure**	**Interpretation**
KMO≥0.90	Marvellous
0.80≤KMO<0.90	Meritorious
0.70≤KMO<0.80	Average
0.60≤KMO<0.70	Mediocre
0.50≤KMO<0.60	Terrible
KMO<0.50	Unacceptable

medRxiv preprint doi: https://doi.org/10.1101/2023.12.05.23299470; this version posted December 5, 2023. The copyright holder for this preprint (which was not certified by peer review) is the author/funder, who has granted medRxiv a license to display the preprint in perpetuity.
It is made available under a CC-BY 4.0 International license .

PCA: Factor analysis, pcf

factor $xlist1, pcf factors(1)

Creating binary index

xtile lol=lol_index, nq(2)

Creating the index for loss of livelihood as a continuous variable.

gen lol_index = .
factor $xlist1, pcf factors(1)

Creating a Kernel density estimate to check the distribution of loss of livelihood index.

gen lol_index = .
factor $xlist1 , pcf factors(1)
predict temp // SPECIFY REGRESION OR BARTLETT OPTION
replace lol_index = temp
drop temp
kdensity lol_index

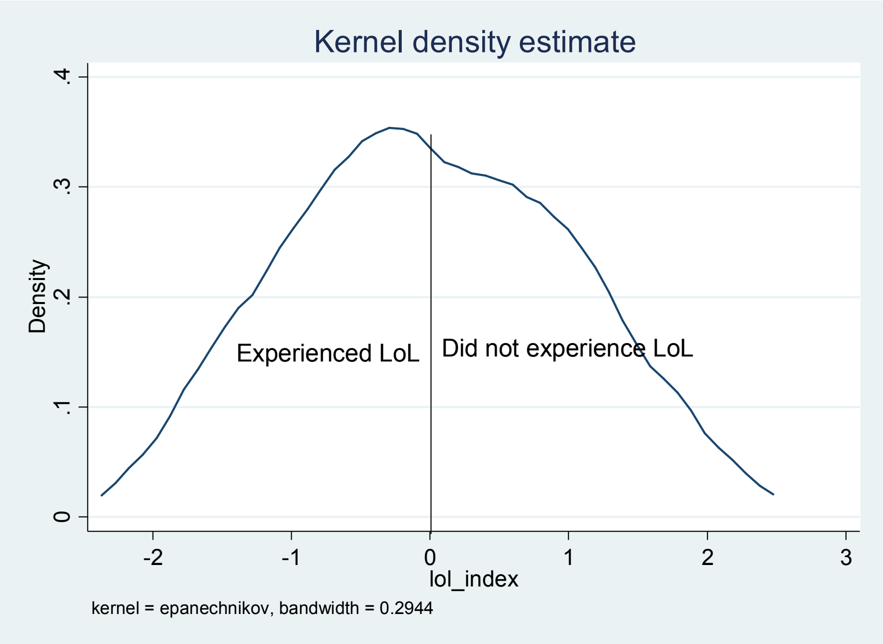

Creating the binary variable for loss of livelihood

gen lol=.
xtile temp=lol_index, nq(2)
replace lol = temp
drop temp
label define lol 1 "Yes" 2 "No"
label var lol "Loss of livelihood"
labelvalues lol lol
ta lol

Experienced loss of livelihood	Number	(%)
Yes	134	(50.2)
No	133	(49.8)
